# Multimodal artificial intelligence and machine learning in oncology: from data integration to precision cancer care

**DOI:** 10.3389/fdgth.2026.1872286

**Published:** 2026-08-24

**Authors:** Shruthi Suresh, A. S. Parvathy, Megha Raj, Keziya G. S., M. Lakshmi Priya, Rhitam Biswas, Sudha Ramaiah, Anand Anbarasu

**Affiliations:** 1 Department of Bio-Medical Sciences, School of Bio-Sciences and Technology (SBST), Vellore Institute of Technology (VIT), Vellore, Tamil Nadu, India; 2 Department of Biotechnology, SBST, VIT, Vellore, Tamil Nadu, India; 3 Department of Bio-Sciences, SBST, VIT, Vellore, Tamil Nadu, India

**Keywords:** deep learning, digital twins, multimodal artificial intelligence, multi-omics, precision oncology, radiomics

## Abstract

Cancer remains a major global health burden, with approximately 20 million new cases and 9.7 million cancer-related deaths reported globally in 2022. While advances in radiological imaging, molecular profiling, and clinical data have enhanced the interpretation of disease progression, the availability of multiple such modalities still does not meet the needs of a large patient population. This narrative review focuses on the role of multimodal artificial intelligence and machine learning in bridging the gap in interpreting heterogeneous modalities to improve risk prediction, prognostic assessment, and treatment decision-making in precision oncology. Multimodal frameworks such as Pathomic Fusion illustrate how complementary histopathological and genomic information can be integrated for cancer diagnosis and prognostic modeling. Multimodal models have demonstrated potential in virtual biopsy, cancer screening, prognostic prediction, radiotherapy planning, intraoperative guidance, and clinical-trial design using digital twins and synthetic control arms. The major limitations of incorporating multimodal artificial intelligence and machine learning in oncology include data heterogeneity, demographic or institutional biases, and reproducibility challenges that hinder translation. Accordingly, appropriate data-governance strategies, fairness audits, and privacy-preserving approaches such as federated learning should be considered where appropriate. Future progress will depend on the development of standardized benchmarking datasets, robust external validation, seamless integration with electronic health records and picture archiving and communication systems, and the implementation of explainable, secure, and clinically validated multimodal artificial intelligence frameworks that support precision oncology in routine clinical practice.

## 1 Introduction

The burden of incidence, mortality, and disability-adjusted life years (DALYs) of cancer is continuously increasing across different parts of the world and diverse communities (1). GLOBOCAN shows that in 2022, there were nearly 20 million new cancer cases and 9.7 million cancer-related deaths globally (2). There are obvious trends and disparities in the prevalence of early-onset malignancies across the world, which contribute to the increased burden, particularly between 15 and 49 years of age (1). Along with ongoing developments in precision oncology and personalized medicines, early identification through efficient screening programs continues to be a key strategy for reducing the cancer burden and enhancing patient outcomes (3). Cancer is also a major public health problem in India, with future predictions indicating an increasing burden (4). A significant proportion of cancer patients globally still have considerable unmet needs, regardless of advances in clinical oncology and cancer research (5). These unmet needs encompass a range of aspects, including everyday life, physical, psychological, and emotional care, support, information, and health system needs (5). For example, it was shown in a Ugandan study that adult cancer patients have a high burden of unmet needs, which is detrimental to their health status and views of care (6). Similarly, cancer survivors also have unmet physical, social, emotional, and medical needs, irrespective of their stage or type of treatment, in lung cancer survivors (7). Long-term survivors continue to have difficulty accessing information, relationships, emotional well-being, and quality of life. With the emergence of immunological, biological, and precision therapies, patients with advanced cancer often require significant support throughout due to the complexity of their disease and treatment (3). Caregivers also experience considerable unmet needs related to financial, psychological, and informational issues (8). Particularly, the number of community-based cancer-specific survivorship support services is lacking, hence making rural cancer survivors face an enormous burden of unmet needs (9). These multifarious unmet needs must be addressed to improve the quality of life and enhance the delivery of cancer care worldwide.

Artificial intelligence (AI) is continually advancing, presenting new possibilities in oncology for diagnostic, prognostic, and therapeutic applications (10). Because AI can analyze large-scale data and discover previously unknown relationships, it has shown potential to improve the accuracy and effectiveness throughout the cancer care cycle and support multiple stages of the cancer care pathway (11). AI has emerged as an important computational approach in oncology, with applications spanning diagnosis, prognosis, treatment planning, and clinical decision support (12). AI is increasingly being integrated into multiple areas of oncology, particularly pathology, where it supports cancer diagnosis, prognostic assessment, biomarker discovery, treatment selection, and precision medicine (10, 13). AI, including machine learning (ML), deep learning (DL), and radiomics-based approaches, is increasingly being applied in oncology for cancer screening, diagnosis, prognosis, and treatment planning (14). For example, a deep convolutional neural network (CNN) demonstrated diagnostic performance comparable to that of board-certified dermatologists for classifying skin lesions under the study conditions (15). Similarly, in mammogram interpretation, AI outperformed a group of radiologists during screening, with 2.7% fewer false negatives and 9.4% fewer false positives (16). Beyond that, ML-enabled combinations of multi-omics and radiomics have shown potential for diagnosing cancer, predicting treatment outcomes, and identifying novel biomarkers (14). Both diagnosis and outcome prediction have improved with the integration of AI in neuro-oncology, leading to increased diagnostic accuracy, enhanced patient outcome prediction, automation of clinical workflows, and optimized treatment plans. Significant progress in developing AI-powered instruments has been observed in the automation of tumor segmentation, genetic-type classification, risk prediction, evaluation of response to therapy, and computational pathology (17). AI has increasingly been applied across cancer prevention, screening, early detection, diagnosis, and treatment personalization (12). However, all ethical considerations, data-related issues, and algorithmic biases must be addressed to enable the responsible implementation of AI in clinical settings (18).

DL has significantly advanced medical image analysis by enabling automated hierarchical feature extraction from complex imaging datasets, thereby improving disease detection, classification, and prognostic prediction. Recent developments include Bayesian optimization strategies that improve model robustness and hyperparameter selection in CNNs, as demonstrated by Bayesian-tuned ResNet-50 frameworks for automated detection of large-cell lung carcinoma (19). Likewise, CNN-based approaches have been integrated with secure medical image transmission methods to simultaneously address diagnostic performance and data security challenges in clinical environments (20). Beyond oncology, DL has also demonstrated considerable success in neurological disorders, including autism spectrum disorder, where multimodal neuroimaging and clinical datasets have been integrated to improve diagnosis and biomarker discovery (21). These advances collectively highlight the versatility of DL architectures and their growing importance across diverse biomedical applications, providing the methodological foundation for multimodal AI systems in precision oncology.

The transition from unimodal to multimodal AI represents a major shift in how AI systems integrate and analyze heterogeneous information. Multimodal AI models integrate complementary information from different data types, enabling joint representation and analysis that cannot be achieved when each modality is processed independently. Unlike unimodal models, which are typically designed to process a single data type, multimodal frameworks can jointly analyze complementary information from sources such as clinical text, medical imaging, pathology, and molecular data (22). Traditional models are limited by structures that only work with a single type of data, such as transformers for text and convolutional networks for images. Cross-modal alignment during training can facilitate the learning of shared representations and may improve generalization across related multimodal inputs. To enable zero-shot recognition of objects described in natural language, even in the absence of explicit labels, for example, use a distinct learning objective that aligns text and image representations into a shared semantic space (23). Foundation models use large-scale pretraining followed by task-specific adaptation, enabling transfer of learned representations across downstream multimodal tasks. Through cross-modal attention mechanisms, models like LLaVA combine visual encoders with large language models to process both textual and visual inputs simultaneously. Studies have shown that removing interactions among visual tokens significantly degrades object recognition performance, thereby confirming the necessity of multimodal feature fusion. In the real world, multimodal systems may provide significant advantages in difficult fields, such as health care, where the fusion of genomic, medical history, and imaging data may boost diagnostic accuracy. Multimodal AI may improve robustness by integrating complementary information across modalities, particularly when individual modalities provide incomplete or ambiguous information. Despite significant advancements in applying AI to cancer, most published papers either explore multimodal techniques within a narrow clinical context or focus on individual modalities such as imaging, pathology, genomics, or clinical records in isolation. A thorough, up-to-date summary that incorporates recent developments in multimodal data fusion, DL architectures, foundation models, and their clinical applications across the cancer care continuum is still required, as multimodal AI is evolving rapidly. Additionally, rather than being treated in a cohesive framework, significant issues, including data heterogeneity, model interpretability, external validation, bias, regulatory considerations, and clinical implementation, are frequently explored independently. These gaps served as the basis for the current review, which aims to provide a comprehensive overview of multimodal AI strategies used in oncology, highlight recent technological developments, critically examine current constraints, and suggest future paths for integrating these technologies into standard precision cancer care.

**Figure 1:**
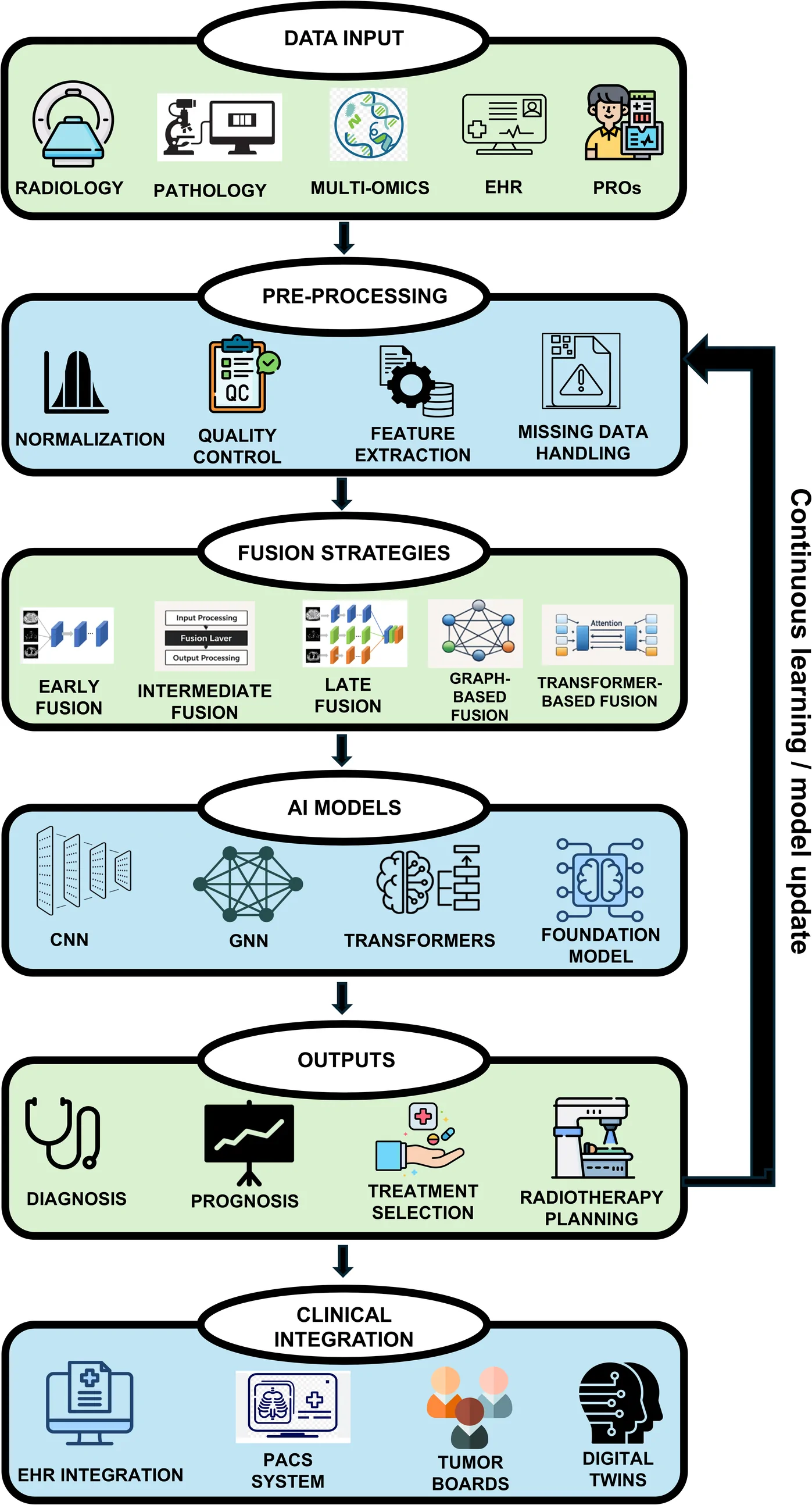
End-to-End workflow of multimodal AI in oncology. This figure illustrates the end-to-end workflow of multimodal AI in oncology, integrating radiology, pathology, multi-omics, EHRs, and patient-reported data. The pipeline includes data preprocessing, multimodal fusion (early, intermediate, and late, as well as graph-based), and AI models such as CNNs, GNNs, transformers, and foundation models. These generate clinical outputs, including diagnoses, prognoses, and treatment plans. Integration with clinical systems (e.g., PACS and EHRs) and feedback loops enables adaptive learning for precision oncology.

The main scope of this narrative review is to provide a comprehensive overview of emerging multimodal AI approaches in precision oncology, with particular emphasis on multimodal data integration, model architectures, clinical applications, translational challenges, and future directions. We discuss recent AI-based methodologies, including radiomics, ML, and DL, as well as approaches that integrate complementary biological and clinical data modalities, including medical imaging, genomics, pathology, and electronic health records (EHRs). We further examine applications of AI across cancer screening and detection, diagnosis and molecular characterization, prognostic assessment, treatment response prediction, and therapy decision support, based on recent literature. Particular attention is given to the potential contribution of these approaches to clinical decision-making and the delivery of more personalized, efficient, and accessible cancer care, while also considering the current limitations and requirements for clinical translation. A schematic overview of the end-to-end multimodal AI pipeline, integrating diverse data modalities with clinically relevant outputs, is presented in Figure 1.

## 2 Methods

### 2.1 Literature search strategy

A comprehensive literature search was conducted to identify relevant studies on multimodal AI and ML applications in oncology. PubMed, Scopus, Web of Science, and IEEE Xplore were systematically searched using combinations of the following keywords: “multimodal artificial intelligence,” “multimodal machine learning,” “deep learning,” “oncology,” “precision oncology,” “radiomics,” “pathomics,” “digital pathology,” “multi-omics,” “medical imaging,” “electronic health records,” and “foundation models.” Google Scholar was additionally searched as a scholarly search engine to identify relevant publications and supplementary literature. Boolean operators (AND/OR) were used to combine search terms where appropriate to maximize the retrieval of relevant literature.

### 2.2 Study selection

This review was conducted as a narrative review. Peer-reviewed articles published between 2016 and 2026 were primarily considered to capture recent advances in multimodal AI for precision oncology. Seminal studies published before this period were also included, where necessary, to provide historical context or to describe foundational concepts. Conference abstracts, duplicate publications, non-English articles, and studies unrelated to multimodal AI or oncology were excluded.

### 2.3 Inclusion criteria and study prioritization

Studies were selected based on their relevance to multimodal data integration, multimodal fusion strategies, AI/ML methodologies, clinical oncology applications, translational potential, and emerging foundation-model-based approaches. Greater emphasis was placed on recent high-quality original research articles, systematic reviews, landmark methodological studies, and investigations reporting external validation, prospective evaluation, or clinically relevant findings. References cited within key publications were also examined to identify additional relevant literature.

## 3 Core AI and ML concepts relevant to oncology

### 3.1 Overview of the ML paradigms

With the objective of improving cancer diagnosis, prognosis, and treatment planning, AI in oncology increasingly relies on ML techniques to assess various biological data such as genomic information, medical records, and medical images. Support vector machines, decision trees, and random forests are some algorithms used in supervised learning that use labeled datasets for prediction tasks, such as categorizing tumors from histopathology images and forecasting clinical outcomes. Precision oncology and evidence-based clinical decision-making have both benefited greatly from these strategies (24). To find hidden patterns and naturally existing clusters, unsupervised learning examines unlabeled data. With the goal of better understanding tumor heterogeneity and facilitating precision oncology, algorithms like K-means clustering are frequently used in oncology to categorize patients or tumors into discrete groups based on molecular or clinical criteria (25). Whilst reinforcement learning has been shown to optimize sequential decision-making, such as adaptive therapy, which aims to optimize subsequent medications based on drug responses to maximize effectiveness, semi-supervised learning can leverage large amounts of unlabeled data together with a smaller amount of labeled data, which may be particularly useful in rare cancers where labeled datasets are limited. In such complex cases, random forests and other ensemble techniques combine multiple models to achieve higher predictive performance (25).

### 3.2 DL, transformers, and foundation models

A modified graph model is integrated into a multimodal graph neural network (GNN) approach for cancer subtype classification, incorporating multiple omics layers to capture intra- and inter-omics interactions and biological relationships accurately. The method has demonstrated improvements in both classification power and interpretability. Also, it demonstrates how GNNs can serve as the organizational mechanism for multimodal integration in oncology by constructing graphs that represent within- and across-gene relationships in expression, methylation, and copy number. Each tumor sample can be more accurately assigned to distinct molecular subtypes, since the framework leverages prior biological knowledge, such as protein-protein interactions and pathway annotations, to inform feature representation learning (26). In general, this approach is well-suited for integrative multi-omics profiling in precision oncology due to the benefits of a graph-based structure that preserves relational information when integrating heterogeneity (26). Furthermore, an overview of multimodal data integration for oncology in the era of DL discusses the combined application of transformers based on large language and vision models, CNNs, and GNNs to combine the imaging, multi-omics, and clinical text data. GNNs can represent relational structures in biological networks and clinical graphs; CNNs can extract task-specific features from radiology and pathology images; and transformers can learn cross-modal attention to bridge and reason across textual and visual modalities. After merging these models into one comprehensive framework, the whole method is capable of producing an end-to-end fusion method by integrating different sources of data like genomic profiles, clinical text in electronic health records, and histopathological slides into a unified predictive model, which could assist research on more accurate and easily accessible oncology diagnostics, prognosis, and therapeutic treatments (27).

### 3.3 Explainability, uncertainty, and robustness

By highlighting clinically significant areas in radiological and histological images, saliency-driven explainable AI strategies enhance the transparency of DL models in oncology. Clinicians can understand model predictions and confirm that diagnostic findings are based on biologically important image features by using techniques like Grad-CAM, Layer-wise Relevance Propagation (LRP), Integrated Gradients, and attention-based visualization. Additionally, quantitative evaluation of saliency maps improves explainable AI's clinical applicability and dependability, facilitating the use of DL models in precision oncology and cancer detection (28). Developing accurate clinical AI systems requires counterfactual reasoning, uncertainty estimates, and model calibration, especially when models are used in contexts that are different from their training data. Predictive performance can be significantly diminished by distributional shifts brought on by differences in patient populations, imaging procedures, or healthcare environments. AI models can detect faulty predictions, increase resilience, and facilitate better clinical decision-making in a variety of clinical contexts by incorporating uncertainty quantification and causal inference (29).

## 4 Major data modalities in oncology

### 4.1 Radiology

In radiology, Computed Tomography (CT), MRI, Positron Emission Tomography (PET), and ultrasonography (USG) are commonly used to identify and monitor tumors in oncology. Applying DL technologies, such as CNNs, to implement end-to-end feature learning, radiomics extracts high-dimensional features, such as GLCM textures and shape characteristics, from these images, thereby improving predictive power in cancer therapy. The radio-genomics model integrating genomics derived from RNA-seq and clinical information of breast cancer patients, along with the radiomics of MRI images (e.g., wavelet-LLH skewness, grey level non-uniformity), builds a nomogram using LASSO-SVM to identify lymph node metastases without surgery [Area Under the Curve (AUC) = 0.82], which has outperformed unimodal models and has justified neoadjuvant therapy selection (30, 31). CT radiomics, along with digital pathology [programmed death ligand 1 (PD-L1) expression] and genomics (MSK-IMPACT mutations), is integrated into a multimodal DL model to assess response to PD-1 blockade in NSCLC. The DyAM (dynamic attention model), built on a combination of imaging and genomics insights, achieved a higher AUC of 0.84 compared to Tumor Mutational Burden (TMB)-only 0.72 in 247 patients, enabling tailor-made therapy (32).

### 4.2 Digital pathology and spatial histology

Digital pathology, a new paradigm in histopathology, enables the entire glass slide to be converted into a computer-readable image using whole-slide imaging (WSI), which provides high-resolution scanning (33). WSI method has become crucial for improving the accuracy of cancer diagnosis, as it automates the extraction of cellular parameters such as morphology, intensity, and texture, as well as cellular- and tissue-level characteristics such as glandular architecture and tissue stroma structure (34). Spatial histology has shown potential to improve WSI by combining spatial information from methods such as spatial transcriptomics and multiplexed immunofluorescence to show how and in which parts of the tissue the proteins and genes are expressed and maintained in a spatial relationship, but visualizing spatial variations can be of diagnostic significance in treatment response, such as to immunotherapy (33, 35, 36). Graphs are a common representation of tissues in graph-based pathological models, where nodes are regions or cells defined by deep features and edges capture spatial adjacency or functional relationships. Using techniques such as coarsening hierarchies and Graph Attention Networks (GATs), which incorporate local-to-global information without relying on spatial regions, improves accuracy over patch-based CNNs for survival prediction and has shown potential to improve interpretability (37, 38).

### 4.3 Genomics, transcriptomics, and other omics

Precision oncology relies on our improved understanding of cancer biology, with all the omics layers (genomics, transcriptomics, and others) now being available. Cancer arises from the accumulation of genetic, epigenetic, and cellular alterations that disrupt normal regulatory processes and promote uncontrolled proliferation and survival (39). These normal processes are altered either by changes in the nucleotide sequence or by changes in the number of copies (copy number variation) of an oncogene or a tumor suppressor gene. Alterations in these genes, combined with modifications in DNA methylation and mRNA expression levels, provide a profile that can classify tumors and even predict treatment response. Experimental evidence in this field indicates that cancer develops through a series of mutations and epigenetic alterations rather than a single one (39–42). Modern technologies for sequencing and gene expression analysis are enabling scientists to produce maps of alterations in samples from many tumors, highlighting important pathways (43, 44). Proteins are key functional effectors of cellular processes, and their abundance and activity may not always correlate directly with corresponding DNA-level alterations (45, 46). This aspect emphasizes the importance of proteomics for targeted drug development. All omics layers help with patient stratification, selection of therapies, tracking the development of drug resistance, and, thus, personalized care (47).

### 4.4 Clinical data, PROs, and free text

It is important to note that often overlooked but useful unstructured information, including clinician notes, policies, and patient-reported outcomes (PROs), is as valuable in clinical data as simpler, structured information such as stages, coded diagnoses, and lab results. Related systematic investigations confirm that adding narrative improves performance in studies on risk factors, case finding, drug safety surveillance, and clinician decision support (48). The free-text narrative from EHRs contains symptomatic patterns, situational details, and clinician logic that would be impossible to translate into codes alone. Although not yet systematically acquired and analyzed, PROs are accepted as the central endpoint in trials and guidelines. They capture patient-centered symptoms, quality of life, and functional status that are not measured by markers or lab data (49). Together, structure, narrative, and PROs would contribute the best possible information on the state of the disease and treatment effects.

### 4.5 Emerging data sources

High-resolution, time-resolved anatomical and functional information is delivered by real-time images acquired during radiation therapy, providing the basis for online and dose-painting treatment adaptations to better control target coverage and protect organs-at-risk. With such methods, it is possible to use soft-tissue volume-based images to track anatomical changes, increase the precision of treatment planning, and perform direct online or offline adaptive replanning (50). The possibility of tracking aggressive sub-regions within the tumor using quantitative functional PET imaging has enabled the development of dose-painting strategies. PET imaging allows radiation dose escalation to radioresistant subregions on a voxel-by-voxel basis while minimizing dose delivery to less aggressive tumor regions. Furthermore, biologically guided adaptive dose painting strategies combined with frequent imaging during radiotherapy have demonstrated the potential to improve the therapeutic ratio by adapting treatment to temporal changes in tumor biology (51, 52). A summary of key oncology data modalities, associated features, and their roles in AI-driven applications is provided in Table 1.

**Table 1:** Major data modalities in oncology and their roles in multimodal AI.

Modality	Data type	Feature extracted	AI techniques	Clinical use	References
Imaging	CT, MRI, PET, x-ray, and radiomic imaging data	Tumor size, shape, texture, heterogeneity, and quantitative radiomic features	CNN	Diagnosis and discovery, prognosis, and type of tumor	(32)
Pathology	Histopathological images including WSI	Cell morphology, tissue architecture, and spatial relationships within the tumor microenvironment	CNN	Classification of tumor (grade and subtype), prognosis	(10, 34)
Genomic	Gene expression, DNA mutation, miRNA, DNA methylation, and RNA-seq data	Gene mutations, gene expression patterns, molecular signatures, and epigenetic alterations	GNN, ML models	Prognosis at the molecular level, personalized medicine	(26, 63, 64)
EHR	Clinical notes, patient history, patient profile, and laboratory results	Patient demographics, clinical history, laboratory findings, and longitudinal clinical information	NLP, ML	Prediction of risk, treatment strategy, and decision-making	(48, 66, 68)

## 5 Multimodal data representation and fusion strategies

**Figure 2:**
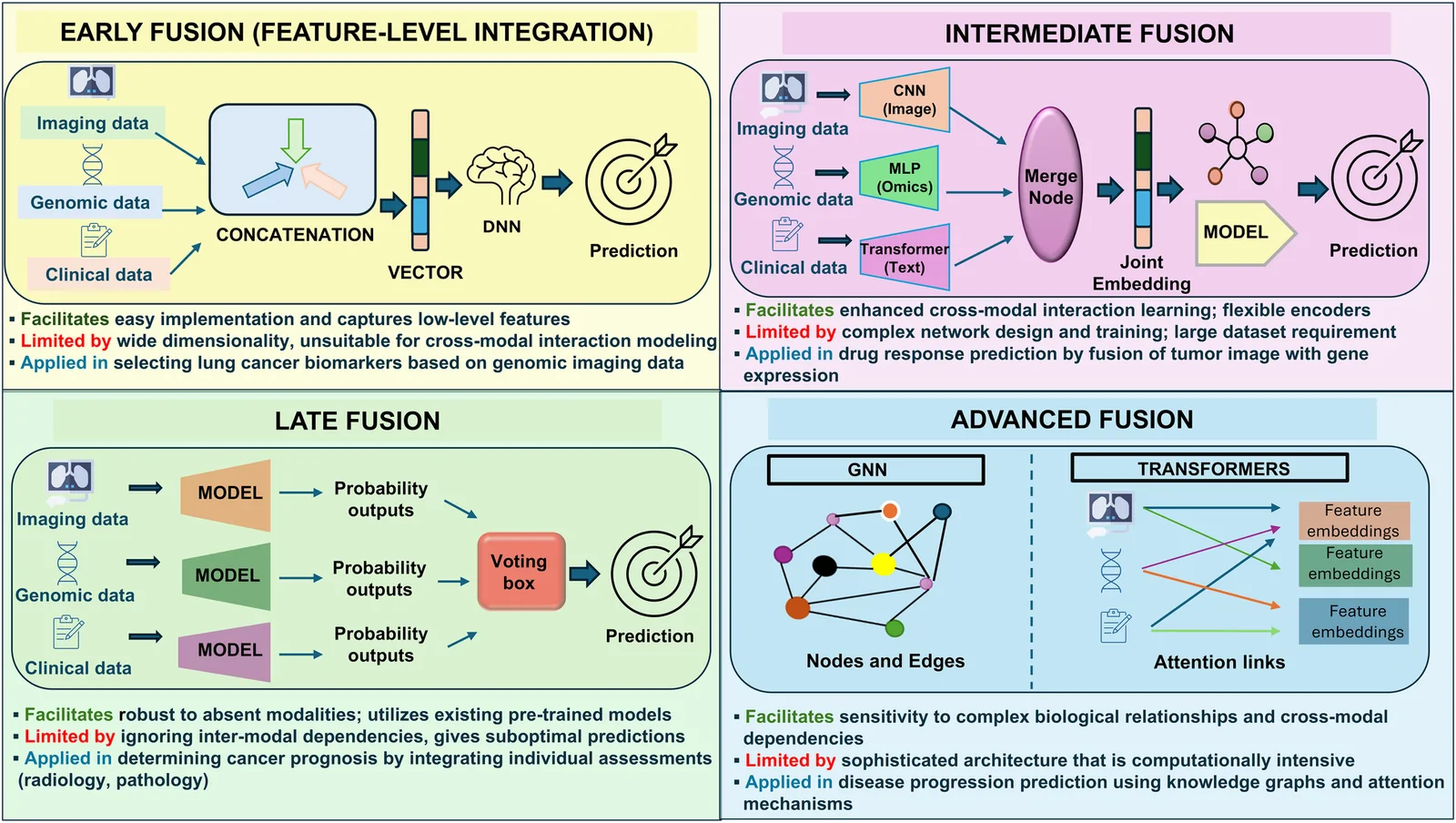
Multimodal data fusion strategies in oncology: conceptual framework and comparative overview. This figure presents a comparative overview of multimodal fusion strategies in oncology. Early fusion combines raw features, intermediate fusion integrates learned representations, and late fusion aggregates predictions from independent models. Advanced approaches, including graph-based and transformer-based methods, capture complex cross-modal relationships. The figure highlights key differences, advantages, and applications of each strategy in cancer diagnosis, prognosis, and treatment prediction.

Imaging technology has long been established as a tool for understanding cancer and its management in clinical practice. While the emphasis was mainly on tumor morphology, imaging could not fully explain mechanisms at the molecular level until the emergence of radiogenomics, the integration of radiomics and genomics to understand tumor heterogeneity across various cancers and improve diagnosis and prognosis. Multimodal data integration can be achieved through early, intermediate, and late fusion strategies, as well as advanced graph- and transformer-based approaches, as illustrated in Figure 2.

### 5.1 Early fusion and feature-level integration

Multimodal models have been investigated for risk assessment and predicting treatment response in cancer patients. They combine diverse data modalities, including imaging, genomics, multi-omics, histopathology data, and other clinical data (53). Multimodal data can be integrated at different stages of the modeling pipeline, commonly categorized as early, intermediate, and late fusion. Early fusion is a relatively straightforward integration strategy in which features from multiple modalities are concatenated into a common input representation before model training (54). Early integration of radiogenomics with clinical and imaging data is also promoted in radiomics using multivariable Cox models. Compared with predictions from single modalities, early fusion yielded higher predictive performance (55). This kind of model is used to predict tumor characteristics at the molecular level by integrating gene expression data and radiomics to improve prognosis (56). The application of early fusion is also found in multi-omics, where gene expression, DNA methylation, and microRNA (miRNA) expression data are integrated at the feature level and processed using a Mixture-of-Experts (MoE) model (57). In essence, these examples indicate that early fusion is most beneficial when the datasets are complete and aligned. Although feature-level integration has been associated with improved predictive performance, inconsistent data preprocessing, small sample sizes, and model complexity due to a large feature space pose challenges to multi- and cross-model integrations.

Early fusion integrates heterogeneous modalities at the feature level by concatenating raw or engineered features before model training, allowing the model to learn complementary information simultaneously. This strategy is particularly suitable for datasets in which imaging, genomic, and clinical variables are consistently available and well aligned. Its principal advantage lies in its conceptual simplicity and ability to capture cross-modal interactions during model training. However, directly concatenating heterogeneous features often yields high-dimensional feature spaces, increasing the risk of overfitting, particularly in relatively small oncology datasets. Moreover, early fusion is sensitive to missing modalities and differences in data preprocessing, limiting its robustness in real-world clinical settings. Consequently, although early fusion has demonstrated encouraging performance in several retrospective studies, its widespread clinical implementation remains constrained by challenges in data harmonization and external validation.

### 5.2 Intermediate fusion and joint representation learning

Intermediate fusion first learns modality-specific representations and subsequently integrates these representations within a shared latent space. This approach enables the model to capture cross-modal interactions while preserving information specific to each modality (58). The representation can be generated via similar neural networks, yielding a homogeneous network, or via various networks that serve heterogeneous models (59). Concatenating PET and CT images at various stages of feature extraction has yielded better results than unimodal approaches for categorizing non-small cell lung cancer (NSCLC) subtypes, enabling non-invasive identification and prognosis (58). Data modalities such as clinical records, WSIs, and molecular studies are fused within a transformer framework such as SurvMBC to predict HER2-positive breast cancer survival rates (60). Compared with direct feature concatenation, intermediate fusion can more effectively model interactions between heterogeneous modalities. However, the lack of appropriate datasets and the computational complexity of training systems to identify modality-level interactions for accurate predictions still hinder the use of intermediate fusion of modalities in oncology.

Unlike early fusion, intermediate fusion first learns modality-specific representations before integrating them into a shared latent space, enabling more effective modeling of complex interactions between heterogeneous data types such as radiology, histopathology, genomics, and clinical records. This approach preserves modality-specific information while improving cross-modal feature learning, making it particularly suitable for multimodal precision oncology applications. Nevertheless, intermediate fusion architectures are computationally intensive, require large, annotated datasets for effective training, and often exhibit limited interpretability. Although they generally outperform simpler fusion methods in research settings, prospective multicenter validation and demonstration of clinical utility remain limited.

### 5.3 Late fusion and decision-level integration

The increasing heterogeneity of cancer types and the growing number of available data modalities have motivated the development of flexible strategies for multimodal integration. Late fusion models have been proposed for their ability to train each model independently and then combine the resulting predictions into an ensemble. Late fusion ensembles can accommodate missing modalities, integrate heterogeneous data sources with limited inter-modal correlation, and reduce the potential dominance of high-dimensional modalities (54). This strategy has been reported to be successful in NSCLC subtyping by separately training different modalities, such as histopathology and omics, to produce class-based probabilities, which are integrated to decide on a learned weighted sum. The late fusion of RNA sequencing, miRNA sequencing, DNA methylation, copy number, and WSI using probability-based methods achieved a higher F1 score and AUC, indicating greater precision (61). Similarly, large-scale datasets such as TCGA, which integrate omics, histopathology, and clinical data from numerous breast cancer patients, are used for predictions within a systematic framework driven by cross-validation and held-out tests to avoid biased risk assessments of the sample population. Late-fusion models aid navigation in computational oncology research and improve prognosis (62).

Late fusion combines predictions generated independently by modality-specific models rather than integrating features directly. This modular architecture offers greater flexibility, facilitates independent optimization of each modality, and is particularly advantageous when certain data modalities are unavailable or inconsistently collected across institutions. However, because interactions between modalities are considered only at the decision stage, late fusion may fail to exploit complex biological relationships that could improve predictive performance. Consequently, although late fusion can be advantageous for heterogeneous clinical datasets, its performance may vary across applications and may not fully capture the cross-modal interactions learned by intermediate or joint representation approaches.

### 5.4 Graph-based and topology-aware multimodal models

With the advent of novel approaches such as ML, DL, computational biology, and systems biology, integrating network models in oncology has become simpler, facilitating the selection of appropriate modeling tools for analyzing large datasets. GNNs were combined with DL and applied in oncology to analyze and synthesize graphs derived from molecular structures and biological data networks. With the ability to incorporate both current knowledge and data-driven learning, models such as Bayesian networks and probabilistic directed acyclic graphs are widely employed in cancer research (63). Multi-omics modalities such as gene expression, miRNA, and DNA methylation, obtained from TCGA BRCA and pan-cancer cohorts, were used to build a GNN that employs GCNs and GATs to extract features (26). Graphs based on these features are also used to form individual patient graphs from subtype and severity datasets using person-to-person networks, integrated with spectral clustering or multiple-kernel learning based on similarity matrices. Both methods yielded precise predictions with higher F1 scores than those from other modalities (64). A recent review by A. Muneer et al. comprehensively summarized the evolution of multimodal cancer data integration from classical ML algorithms to DL and foundation models. Their work highlighted the growing importance of multimodal representation learning, large-scale pretraining, and foundation architectures in improving biomarker discovery, molecular subtype classification, treatment response prediction, and precision oncology, supporting the rapid transition toward generalizable multimodal AI systems (65).

Graph-based fusion represents multimodal biomedical data as interconnected graphs, enabling explicit modeling of biological relationships among genes, proteins, signaling pathways, cells, and patients. Compared with conventional fusion strategies, GNNs are particularly effective at capturing complex relational dependencies that are difficult to represent using feature concatenation alone. These methods have shown considerable promise in multi-omics integration and tumor microenvironment analysis. However, their performance depends heavily on the availability of accurate biological network information, and their substantial computational complexity, limited interpretability, and lack of prospective clinical validation currently restrict widespread clinical implementation.

### 5.5 Large multimodal models and foundation architectures

Generating clinical reports from medical imaging modalities such as x-ray, CT, and MRI is an important component of clinical workflow and can be time-consuming for clinicians (66). Foundation models (FM) are advancing in computational pathology to support the diagnosis, treatment, and prognosis of various cancer subtypes. The Multimodal Multidomain Multilingual Foundation Model (M3FM) is an FM trained on patient medical data and designed to acquire knowledge across modalities, languages, and fields (67). For easier access to patient clinical records spanning several departments and systems, an open-source interface known as Harmonized Oncology Biomedical Embedding Encode (HONeYBEE) was launched to troubleshoot integration challenges via FM-based embeddings, producing clinical records represented as vectors (68). Similarly, Multi-modal Self-TAught PRetraining (mSTAR) pathology FM integrates modalities such as clinician reports, gene expression data, and WSI, and efficiently performs several downstream pathological tasks (67, 69). Although foundation models are promising for multimodal applications, current implementations may be limited by image resolution constraints, interpretability challenges, and substantial computational or infrastructure requirements. A detailed comparison of multimodal fusion strategies, including their advantages and limitations, is presented in Table 2. Transformer-based foundation models extend multimodal learning through self-attention and large-scale pretraining, enabling the development of generalized representations that can be adapted to multiple downstream oncology tasks. Compared with conventional multimodal learning approaches, foundation models offer improved scalability, transfer learning capability, and the ability to integrate diverse biomedical modalities within a unified framework. Nevertheless, these models require massive datasets, substantial computational infrastructure, and rigorous external validation. Furthermore, concerns regarding interpretability, demographic bias, and reproducibility must be addressed before foundation-model-based systems can be routinely deployed in clinical oncology.

**Table 2:** Comparison of multimodal data fusion strategies.

Fusion type	Method	Advantages	Limitations	Application	References
Early fusion and feature-level integration	Data integration of multiple modalities into a single input vector (e.g., Radiology images+clinical data)	Improved prediction of tumor characteristics, concatenation of multi-omics using models like MoE	Lacks consistent data pre-processing, large data, and a sample size for accurate predictions	Tumor characteristics prediction, integration of multi-omics	(56, 57)
Intermediate fusion and joint representation learning	Independently extracts features from each modality using joint representation learning, such as neural networks and transformers like SurvMBC	Effective modeling of cross-modal interactions and potential improvement in predictive performance	Computationally intensive, with difficulty in performing modality-level interaction modeling	Subtype classification in cancer subtypes, such as HER2-positive breast cancer and NSCLC, improved multimodal prediction models	(58, 61)
Late fusion and decision-level integration	Trains model independently with subsequent integration of predictions using probability-based fusion, weighted ensemble	Robust in handling missing modalities in heterogeneous data, avoiding domination within modalities	Limits interaction between modalities, increased dependence on the performance of the individual model	TCGA-multimodal prediction for NSCLC subtype classification improves the prognostic performance	(61)
Graph-based and topology-aware multimodal models	Employing graph neural networks such as GCN, GAT, and network-based models to concatenate multi-omics with biological relationships	Preserves biological relationships and can improve predictive performance and interpretability	Computationally intensive with complexity in modeling and scale expansion	Enhanced subtype classification using multi-omics, similarity networks among patients, and precision oncology	(26, 64)

Overall, no single multimodal fusion strategy is universally optimal for all oncology applications. Early fusion provides computational simplicity but is sensitive to missing data and high-dimensional feature spaces. In contrast, intermediate fusion enables richer cross-modal representation learning at the expense of greater computational complexity. Late fusion offers increased flexibility and robustness for heterogeneous clinical datasets but may not fully exploit interactions between modalities. Emerging graph-based and foundation-model approaches represent the current frontier of multimodal AI by capturing complex biological relationships and contextual information; however, their broader clinical translation will require improvements in interpretability, standardized benchmarking, prospective multicenter validation, and reproducibility.

## 6 Data alignment, curation, and benchmarking

Integrating multiple modalities, including large-scale datasets, is a major challenge due to their heterogeneity. These modalities range from clinical data to highly complex radiologic and molecular genomic data. The predictive performance of multimodal models depends in part on the effective alignment and harmonization of heterogeneous datasets.

### 6.1 Cohort assembly and multi-institutional datasets

While data fusion is essential for processing raw features and making predictions by comparing interrelated information from each dataset, preprocessing is also necessary to address factors that hinder model efficiency, such as noise, bias, and gaps due to missing data points (57). In addition, to determine the cohort assembly, the identification and aggregation of patients who share similarities in molecular and clinical data are necessary, requiring data from multi-institutional EHRs and biobanks. Numerous cancer datasets are available through centralized repositories. Examples include NIH-supported resources such as The Cancer Imaging Archive (TCIA), Genomic Data Commons (GDC), and Proteomic Data Commons (PDC). Although sources such as TCGA incorporate large datasets of patient data, there is a need to extend pan-cancer multimodal models to highlight the challenges of cohort assembly from diverse sources at different scales (27).

### 6.2 Modality alignment and missing data

Fusion models often encounter the variability and lack of structure in data when processing modalities such as imaging (e.g., radiology scans and WSIs) and molecular and clinical data. They are often spread across modalities because they are processed separately, necessitating a specialized model to standardize and integrate patient-level data into scalable frameworks. HONeYBEE is one such framework that helps produce patient-specific embeddings from clinical data (both structured and unstructured), WSIs, radiomics, and molecular data. It primarily relies on modality concatenation and Kronecker product fusion strategies to derive patient data embeddings, even when certain datasets are absent. Thus, it accelerates downstream tasks in oncology by improving prediction, pan-cancer classification, and patient similarity search for cohort assembly (68).

### 6.3 Dataset shift, external validation, and benchmarking initiatives

Although multimodal models are trained on specific diseases or cancer subtypes, this often leads to variability in experimental data, reducing their generalisability due to a lack of external validation. This shift in robustness may arise from variations across cohorts, institution-specific data and collection protocols, and temporal or treatment-related patterns. To standardize performance results, a benchmark framework such as SurvBoard was used to select experimental designs and harmonize experimental design choices. This facilitated single- and pan-cancer comparisons of data models and the evaluation of patient data with absent modalities. The model often outperformed DL programs in estimating the survival function (70).

## 7 Screening and early detection

With multimodal artificial intelligence (MMAI) integrating medical data across sources, predicting high-risk cases, prognoses, and patient lifestyle profiling has become more efficient. These algorithms have the potential to predict various cancer subtypes, such as multimodal frameworks that integrate radiomic and demographic information for cancer risk prediction (71).

### 7.1 Imaging-plus-clinical multimodal screening

The concatenation of modalities, such as medical imaging (CT and MRI), clinical data (EHRs), patient demographics, and molecular profiles via AI/ML multimodal models, plays a key role in the screening and early detection of cancer compared with unimodal models. Advanced DL models, such as CNN-transformer hybrids, facilitate efficient data extraction from heterogeneous sources. In their study, Qian et al. described multimodal approaches that integrate complementary clinical and imaging information for cancer risk assessment, including a breast cancer study involving more than 5,000 patients that combined clinical metadata, mammographic imaging, and trimodal ultrasound (71, 72). The reported findings highlight the potential of multimodal AI for population-level cancer risk prediction and screening. This substantially reduced errors, such as false positives, while effectively highlighting mild, high-risk lesions often missed by standard procedures. The impact of such studies reveals the potential of these multimodal models to help prevent overdiagnosis and reduce disparities among population cohorts. Hence, they promote the efficacy of early intervention and alleviate the worldwide cancer burden.

### 7.2 Multimodal AI in risk prediction and early diagnosis

Early and accurate detection of breast and lung cancers is associated with improved clinical outcomes and can facilitate earlier intervention, which can be achieved via DL models that can screen for mild tumor features on imaging. Although not strictly multimodal, AI-assisted colonoscopy provides an example of how AI can improve cancer-related detection workflows; a recent prospective study reported an increase in adenoma detection with AI guidance (73). For feature extraction to make risk predictions, multiple techniques are employed, such as the AstraZeneca-artificial intelligence (AZ-AI) multimodal pipeline, a Python library that preprocesses and reduces the dimensionality of unimodal and multimodal datasets. In comparison, multimodal models trained via late fusion have outperformed unimodal models in 24 cancer subtypes, improving precision oncology. These models are trained for heterogeneous datasets, giving better overall survival predictions (74).

### 7.3 Population-level deployment and health-system integration

Dellamonica et al. highlight the importance of MMAI in population screening, which requires integrating histopathology, multi-omics, and clinical records, yielding more robust prediction and risk assessment than unimodal models. The framework they have devised presents epidemiological MMAI, making cancer risk predictions using UK Biobank cohorts and achieving an ROC-AUC of more than 0.85, thereby facilitating targeted oncology interventions, such as prioritizing high-risk categories for imaging. Eisemann et al. report the incorporation of AI into German mammography screening for over 460,000 women. AI successfully triaged 56.7% of cases as normal, avoided unnecessary radiology studies by 43%, and increased breast cancer detection rates (BCDR) by 17.6% (75). These studies are instrumental in understanding the impact of AI/ML-driven multimodality on public healthcare, which significantly reduces costs and helps fulfill the World Health Organization (WHO) screening goals for the year 2030. Regulatory agencies such as the U.S. Food and Drug Administration (FDA) have developed regulatory frameworks for AI-enabled medical technologies, although implementation and regulatory requirements vary across jurisdictions.

## 8 Diagnosis, grading, and molecular subtyping

**Figure 3:**
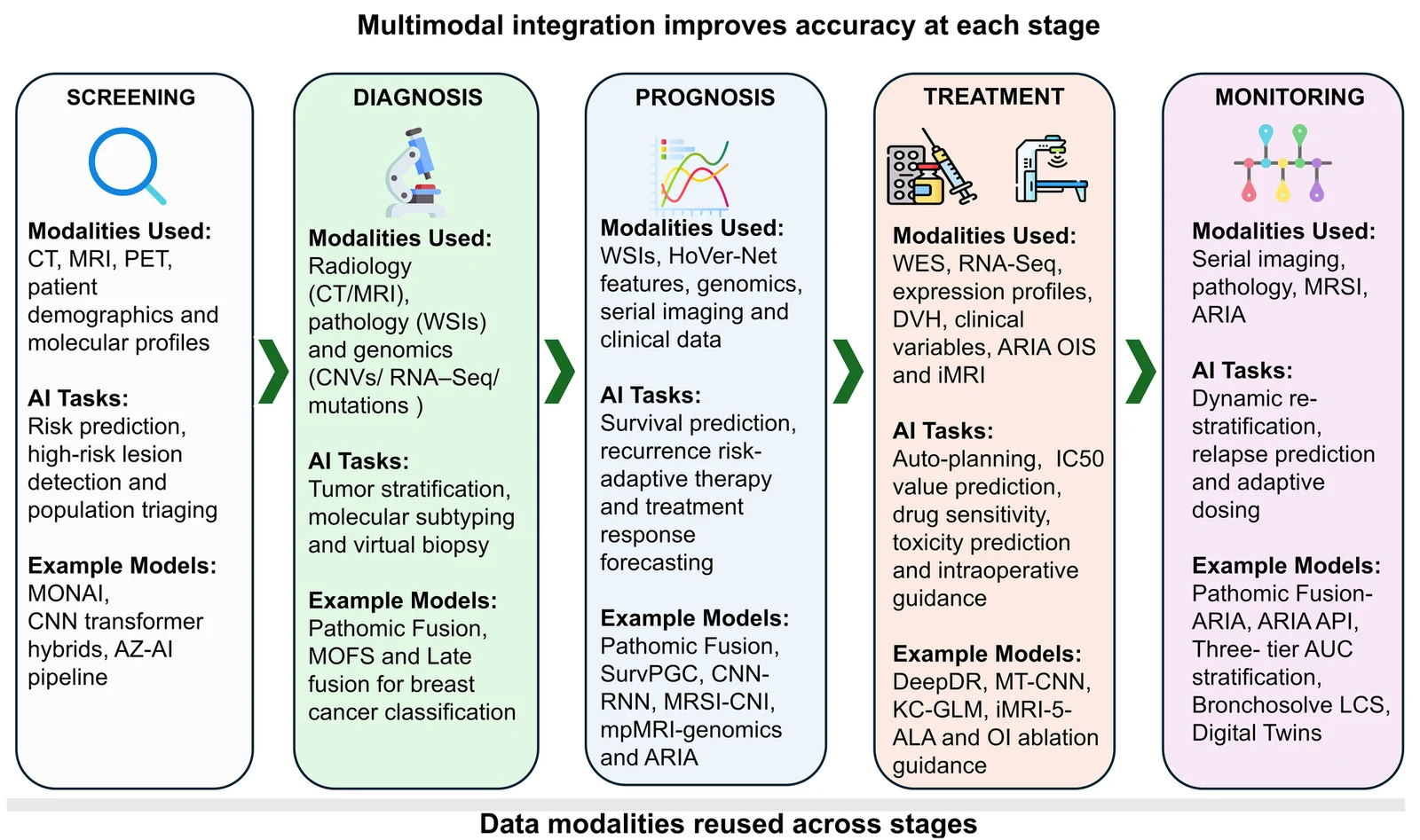
Applications of multimodal AI across the cancer care continuum. This figure illustrates the application of multimodal AI across the cancer care continuum, including screening, diagnosis, prognosis, treatment, and monitoring. By integrating imaging, pathology, genomics, and clinical data, multimodal models enhance risk prediction, tumor classification, survival analysis, treatment optimization, and relapse monitoring. This framework highlights the role of multimodal AI in advancing precision oncology and improving clinical outcomes.

The core limitations of traditional methods in oncology, such as invasive biopsies and data analysis of unimodal data silos, can be addressed by multimodal techniques that integrate diagnosis, grading, and molecular subtyping, fusing diverse inputs like imaging, genomics, pathology, and molecular subtyping for better precision and speed. The role of multimodal AI across different stages of cancer care, from early detection to post-treatment monitoring, is shown in Figure 3.

### 8.1 Radiology-pathology-genomics fusion for tumor characterization

The combination of radiology, pathology, and genomics represents a multimodal approach to tumor stratification (76). Combining the radiological images from equipment like MRI and CT, histological WSIs, and genomic data comprising Copy Number Variations (CNVs), RNA sequencing expressions, mutations, etc (77). Radiology is a major method used in oncology to visualize cancer at macroscopic levels. CT, MRI, and x-rays are used to create three-dimensional images that help diagnose and characterize a variety of diseases, including tumors, and predict treatment responses (78). Pathology provides microscopic-level detail of histology and cell characterization using techniques such as WSIs for various cancers. Genomics investigates the structure, function, organization, and variation of an organism's genome. Integrating these complementary data sources enables feature extraction across multiple biological scales and facilitates the identification of relationships that may not be apparent from individual modalities. Multimodal fusion models can be applied at different stages of tumor subtype classification. Early fusion consists of raw features from all models into a single representation before model training. Intermediate fusion comprises features that are first processed separately and then merged, such as multimodal fusion subtyping (MOFS), which integrates data from MRI, histology, and proteogenomics in glioma patients. Pathomic Fusion is one such example of an internally validated multimodal fusion model evaluated in glioma and renal cell carcinoma classification using histology and genomic data from TCGA, for which external validation and prospective clinical assessments remain limited (77). Late fusion allows predictions from different modalities to be combined to create a hierarchical model, such as combining histopathological images and gene expression profiling for breast cancer classification. Attention-based mechanisms are increasingly used in intermediate and late fusion architectures to model cross-modal interactions and provide attention maps that may support model interpretation.

### 8.2 Virtual biopsies and non-invasive molecular profiling

Virtual biopsy is a non-invasive computational technique for extracting diagnostic, molecular, and/or histopathological information from medical images such as MRI, CT, and ultrasound (79). The extraction is performed using radiomics, a quantitative approach for analyzing texture, shape, and intensity features in the field of AI (80). Such approaches may reduce the need for invasive sampling in selected applications, although their ability to replace tissue biopsy remains investigational. Virtual biopsies may offer more comprehensive, rapid, and repeatable results than invasive biopsies. The major steps in radiogenomics include image acquisition through routine MRI/CT scans that can be missed by physical biopsies due to sampling error, followed by radiomics to encode underlying information from images, and finally the computational modeling comprising of ML (genetic algorithms and affinity propagation) and DL tools (feature selection and extraction), along with adequate training tools and training and validation of data or samples can be performed using the TCGA and TCIA databases, along with hospital cohorts (81). Some of the actionable markers predicted by radiogenomics include isocitrate dehydrogenase (IDH) genes, assessed using contrast-enhanced MRI imaging, such as T1, Fluid-attenuated inversion recovery (FLAIR), and Apparent diffusion coefficient (ADC) maps (82). However, reproducibility challenges such as scanner heterogeneity, feature selection instability, and limited prospective validation constrain clinical adoption. Other biomarkers, such as PD-1 and PD-L1 expression, are used to inform immune-checkpoint inhibitor therapy selection in selected cancers, including NSCLC, renal cell carcinoma, bladder cancer, and head and neck cancers. PET, along with CT scans, is used to investigate immunotherapy eligibility based on these markers, although these models remain investigational (83). A few challenges that limit the application of virtual biopsy in clinical practice include reproducibility issues, high data dimensionality due to scanner/vendor differences, sampling bias, and computational barriers (84).

### 8.3 Tumor microenvironment and spatially resolved multimodal models

The tumor microenvironment (TME) comprises immune cells, stromal fibroblasts, vasculature, and extracellular matrix. It drives immunotherapy response, metastasis, and resistance (85). Traditional genomic analysis methods often lose spatial context because they are not suitable for capturing gene expression profiles and spatial location simultaneously (86). Spatially resolved multimodal models fuse techniques such as computational histopathology, multiplex immunofluorescence (mIF), and spatial transcriptomics to reconstruct cellular neighborhoods and molecular gradients at sub-cellular resolution. Spatial transcriptomics profiles RNA *in situ* and can be complemented by single-cell RNA sequencing (scRNA-seq), providing spatial context for cells and helping with cell type identification and analysis. Slide-Seq, 10× Visium, sequential fluorescence *in situ* hybridization (Seq-FISH), MERFISH, STARmap and GeoMX Digital Spatial Profiler are a few examples of the same (87).

Local structures within the TME are key factors in tumor development and treatment response. Spatial Profiling of Tumors by Leveraging Imaging and Transcriptomics (SPoTLIghT) is a computational tool that can be applied to hematoxylin and eosin (H&E) slides to derive tumor architecture. This is an alternative approach that mimics a pathologist's workflow for identifying tumor-infiltrating lymphocytes (TILs) (88). H&E imaging is otherwise called digital pathology. Advances in AI and ML enabled the combination of mIF and H&E staining. This model has shown potential to improve comparative diagnosis and therapeutic decision-making by providing data from both techniques. Other multiplex imaging techniques are multiple immunohistochemistry (mIHC) and imaging mass cytometry (IMC). As the name suggests, multiplex techniques, rather than traditional IHC and cytometry, allow the simultaneous analysis of multiple markers (89).

## 9 Prognosis and outcome prediction

Multimodal AI/ML helps with prognosis and outcome prediction by shifting clinical decision-making toward personalized approaches, moving from population-level statistics to individual risk stratification. This is done by integrating complementary signals such as imaging, genomics, pathology, and clinical data to predict survival, recurrence, response, and medication toxicity.

### 9.1 Multimodal survival prediction

Multimodal survival prediction integrates results from histology, genomics, and clinical data using DL mechanisms to predict individual risk stratification, demonstrating improved prognostic performance compared to traditional unimodal systems in several cancer-specific cohorts, but generalization needs further external validation (90). Data from histopathology include CNN-extracted features from WSIs, as seen in HoVer-Net, for tumor or stroma segmentation (91). Genomic data include mutations, CNVs, and non-coding RNA sequences such as miRNA and long non-coding RNA (lncRNA) for patient prediction; meanwhile, clinical data include age, tumor stage, and treatment history. Major examples of such models include Pathomic Fusion, a hybrid multimodal fusion that integrates the cell graph representations from HoVer-Net detection with genomic profiles for glioma and renal cell carcinomas. Pathomic Fusion achieved a 6.31% improvement over the WHO paradigm for glioma survival prediction, reaching a C-index of 0.826 (77). SurvPGC is another model that uses late fusion to integrate pathology images with genomic and clinical data to predict the prognosis of various cancers (92). Such models could potentially inform risk-adaptive treatment strategies, although clinical implementation requires prospective evaluation and treatment-effect validation (93). AI-driven dosing is an active area of research, with a limited number of regulatory-cleared AI tools for imaging and workflow tasks, but AI systems to prescribe dosing are under evaluation.

### 9.2 Recurrence, metastasis, and response prediction

Multimodal models predict relapse, patterns of spread, and the effects of radiotherapy or chemotherapy across cancers like glioblastoma by integrating outcomes from multiparametric MRI, outperforming conventional uniform expansion-based target definitions in terms of specificity and sensitivity (94). Recurrence prediction for five years was done for breast cancer with the help of multiparametric magnetic resonance imaging (mpMRI) and genomics using an early fusion architecture (95). Another well-known example is Pathomic Fusion, which forecasts glioma relapse using WSI cell graphs and RNA sequences. The metastatic progression of NSCLC can be comprehended through a series of CT scans in combination with CNN-RNN (recurrent neural network) analyses. Serial longitudinal imaging using CT scans helps assess dynamic responses in the body (96). Glioma recurrence predictions were explored by evaluating Magnetic Resonance Spectroscopic Imaging (MRSI) data paired with the Choline-to-NAA index (CNI) (94). The utility of these methods lies in predicting relapse, metastasis, and drug response in the above-mentioned cancers. ARIA is a Varian Oncology Information System, widely used in radiotherapy centers across the United States. It manages all patient data and provides a fused multimodal model review across imaging modalities such as CT, MRI, and PET. Customized ARIA workflows may help improve workflow efficiency and support more informed treatment decisions, but direct evidence that they reduce dose toxicity or improve dose efficiency remains limited (97).

### 9.3 Stratifying patients for follow-up and survivorship care

Multimodal AI may support post-treatment patient surveillance by integrating pathology, serial imaging, and MRSI data to generate dynamic risk scores for each patient, thereby personalizing the intensity of follow-up. Usually, the three-tier stratification is low-, intermediate-, and high-risk based on predicted recurrence probabilities or risk scores, with thresholds derived from cohort-specific calibration and validated through Kaplan–Meier survival analysis (98). Such risk stratification may support more individualized surveillance and treatment planning. The treatment options may also differ across strata. In principle, models such as Pathomic Fusion could be integrated with oncology information systems such as ARIA to support adaptive radiotherapy workflows; however, automated dose adjustment during treatment remains conceptual and needs external and prospective validation.

## 10 Treatment selection, theranostics, and adaptive care

Multimodal AI has the potential to recommend personalized therapy selection by integrating outputs from genomics, pathology, imaging, and clinical data; however, most applications are currently at the research stage and require further validation before use. AI predictions can be considered more reliable for guided therapy, theranostics, and adaptive care across various cancers through methods such as tumor stratification and tailored survivorship, with a need for external validation tests.

### 10.1 AI-driven precision oncology and drug response modeling

Multimodal AI is contributing to the field of precision medicine by combining diverse biological data, such as whole exome sequencing (WES), RNA-seq, and methylation data, with imaging techniques to predict precision therapies and other treatments more accurately. DeepDR is a research-stage model that predicts the *in vitro* drug response using three DNNs based on cell line genomic profiles. This model forecasts IC_50_ values for 265 drugs commonly used in cancer treatment using mutation and expression profiles (99). In a complementary approach, spatial proteomics and compartment-based transcriptomics were used to analyze the tumor immune microenvironment (TIME) for PD-1 expression in NSCLC patients (100). The Dialogue on Reverse Engineering and Assessment Methods (DREAM) is a crowdsourcing platform for biomedical research that uses challenges to solicit algorithms to solve complex problems in biology, drug response prediction, and precision oncology (101). BeatAML DREAM is a research program that integrates molecular alterations with drug-sensitivity data to improve the generalizability of drug-response models tailored to Acute Myeloid Leukemia (AML) cases, but clinical translation remains ongoing (102). A collaborative effort between the National Cancer Institute (NCI) and the DREAM project yielded NCI-DREAM, a drug-sensitivity predictor. Furthermore, Pancancer Analysis of Chemical Entity Activity (PANACEA) is an extensive repository of dose-dependent cellular responses and detailed molecular profiles following drug treatments in rare tumors such as Gastrointestinal Stromal Tumor (GIST) and GastroEnteroPancreatic NeuroEndocrine Tumors (GEP-NETs) (103). DREAM frameworks improve benchmarking across large datasets, but clinical generalizability still requires independent validation (103). In principle, closed-loop precision therapeutics convert empirical dosing to adaptive care with weekly risk re-stratification through systems such as ARIA API integration, but require regulatory review before clinical adoption, as they are emerging concepts for adaptive care and require prospective validation and workflow-level safety assessment.

### 10.2 Radiotherapy planning and adaptive radiotherapy

Radiotherapy optimization using multimodal AI leverages results from three data streams to generate treatment dosimetric plans and predict toxicity across multiple modalities. The input components include planning CT, Dose-Volume Histogram (DVH), and clinical variables. The key applications include auto-planning using a Multi-Task (MT) network that uses CNNs to predict dose distributions across various RT modalities simultaneously. It uses the patient's CT scan and treatment-protocol dosimetry goals to create a personalized dose distribution for each patient (104). These models are research-stage tools and not yet FDA-cleared RT planning systems. Another model, called the knowledge-constrained generalized linear model (KC-GLM), predicts the toxicity range by integrating DVH constraints with biomechanical factors. This technique is used on datasets for prostate and lung cancer. The model incorporated domain-informed constraints and provided interpretable estimates of toxicity-related variables. This model had advantages, as it integrated three pieces of domain knowledge into its formulation: non-negativity, monotonicity, and adjacent similarity, but their clinical deployment requires external validation (105).

### 10.3 Surgery, interventional oncology, and real-time multimodal guidance

Multimodal AI integrates preoperative scans such as MRI and CT, intraoperative imaging methods such as ultrasound and fluorescence, and clinical data to reduce risk and improve targeted guidance. Glial resection is one of the key applications in which intraoperative MRI (iMRI) is used to improve the Extent of Resection (EOR) in glioma surgery and to detect postoperative neurological deficits (106). Another application is ablation guidance, which can be performed with interventional optical imaging during surgery to provide real-time guidance for complete tumor eradication (107). AI may also be used for lung cancer screening with a closed-loop, fully automated software called Bronchosolve to improve consistency, accuracy, and throughput, and the model achieved a sensitivity of 83.6% and specificity of 86.3% (108).

### 10.4 Multimodal AI in clinical trials and digital twins

Multimodal AI may support clinical-trial design by enabling patient stratification, cohort enrichment, and trial matching using advanced tools. By combining pathology and imaging techniques, high-risk cancer patients can be identified, particularly those suffering from lung and brain cancer. These approaches may support more efficient patient identification and trial matching, although their clinical utility remains under evaluation. Multi-Agent framework-based Knowledge Augmentation and Reasoning (MAKAR) is a multi-agent system, under the search stage, that could improve AI-driven patient matching for clinical trials by enriching trial criteria and evaluating each criterion-related patient condition (109). Digital twins and synthetic control arms have emerged as promising complementary technologies that may improve clinical trial design, patient stratification, treatment simulation, and virtual cohort generation (110). They are individualized *in silico* models and emerging research tools that can replicate patient populations in real time (111). However, these approaches remain largely investigational and should currently be regarded as tools that complement rather than replace conventional randomized clinical trials. Their broader clinical adoption will require robust prospective validation, standardized regulatory frameworks, transparent model development, and careful evaluation of ethical and methodological considerations. Selected clinical and translational applications of AI and multimodal AI across screening, diagnosis, prognosis, treatment planning, and clinical-trial support are summarized in Table 3.

**Table 3:** Selected clinical and translational applications of multimodal AI and related AI/ML approaches in oncology.

Application	Clinical task	Study population/sample size	Modalities/Input data	AI model/Computational approach	Comparator/baseline	Key performance outcome	Validation design	Clinical implementation status	References
Tumor characterization and molecular subtyping	Glioma and renal cell carcinoma diagnosis/prognostic classification	769 patients with histology images	Histopathology/WSI + genomic features	Pathomic Fusion; cell-graph and genomic feature integration	WHO grading and unimodal histology/genomic modelsapproaches	6.31% improvement over WHO grading; C-index = 0.826	15-fold cross-validation using TCGA data; retrospective	Research-stage; external and prospective clinical validation remain limited	(77)
Survival and prognosis prediction	Survival-risk stratification across cancer cohorts	TCGA-LIHC: 354; TCGA-BRCA: 1,035; TCGA-COADREAD: 298 cases	Pathology + genomics + clinical data	SurvPGC, late-fusion multimodal model	SurvPath, PORPOISE, MCAT and other unimodal/multimodal prognostic models	C-index: 0.701 ± 0.054 (LIHC), 0.701 ± 0.057 (BRCA), and 0.676 ± 0.087 (COADREAD), compared with SurvPath values of 0.652 ± 0.054, 0.622 ± 0.038, and 0.644 ± 0.138, respectively	5-fold cross-validation using TCGA cohorts	Research-stage prognostic model	(92)
Clinical-trial matching and patient stratification	Patient-trial matching and enrichment of eligible cohorts	Not specified	Multimodal clinical/pathology/imaging information	MAKAR, multimodal knowledge-augmented reasoning framework	Conventional/manual trial-matching approaches	Improved criterion-level patient matching reported; quantitative clinical performance not established	Research-stage evaluation	Research-stage; clinical utility and prospective validation remain to be established	(109)
Drug-response prediction/precision oncology	Prediction of anticancer drug response	622 cancer cell lines	Mutation and gene-expression profiles	DeepDR, deep neural-network-based pharmacogenomic model	Prior pharmacogenomic prediction models	MSE = 1.96 for log-scale IC50 prediction	Training/testing on 622 CCLE cell lines; external application to 33 cancer types on TCGA data	Preclinical/research-stage; not a clinical treatment-selection system	(99)
Radiotherapy planning	Personalized dose prediction	28 APBI patients; 20 for training/validation and 8 for testing	Planning CT + clinical/treatment-planning information	Multi-task CNN (MT-CNN)	Single-modality/non-multitask dosimetry models; five single-task CNN models	MAPE = 1.1033 ± 0.3627% vs. 1.2386 ± 0.3872% for single-task models; *p* = 0.0003	Train/validation/test split; independent test set of 8 patients	Research-stage planning-support model; not established as routine clinical deployment	(104)
Radiotherapy toxicity prediction	Toxicity prediction in prostate and lung cancer	79 prostate cancer patients; 134 stage III NSCLC patients	Dose-volume histogram (DVH) + biomechanical features	Knowledge-constrained generalized linear model (KC-GLM)	Lasso, elastic-net and fused-lasso models	Higher AUC and improved interpretability compared with comparator models; *p* < 0.03	Patients divided into validation (20) and test (50) cohorts as reported	Research-stage interpretable model; requires external validation	(105)
Intraoperative guidance*	Glioma extent-of-resection and postoperative neurological outcomes	43 patients undergoing iMRI-guided glioma surgery	Intraoperative MRI; comparative imaging information	Intraoperative MRI-guided surgical imaging	Comparative clinical imaging analysis	iMRI associated with more accurate extent-of-resection assessment and lower residual disease than epMRI; SICE: 25% vs. 67.9%	Clinical comparative imaging study	Clinical imaging technology; AI-specific clinical deployment not established	(106)
Lung cancer screening*	Pulmonary nodule detection and stratification	2,358 cases in the training cohort; external cohort of 184 patients across 8 sites	Low-dose CT imaging	Bronchosolve, closed-loop automated AI model	Manual/standard screening workflow	Sensitivity = 83.6%; specificity = 86.3%	External multicenter validation using a US multi-site cohort	Externally validated automated screening model; implementation status requires further clinical evaluation	(108)
Digital twins and synthetic control arms	Trial design, patient stratification and treatment simulation	Not applicable; virtual patient cohorts	Clinical and longitudinal patient data	Digital-twin and synthetic-control-arm approaches	Conventional trial design/standard control arms	Potential to support virtual cohort generation and treatment simulation; no established clinical performance metric	Emerging research; prospective validation required	Investigational; not a replacement for randomized clinical trials	(110, 111)

*These examples are included as clinically relevant AI-enabled technologies discussed in the review but are not necessarily multimodal AI models in the strict sense.

## 11 Technical limitations and biases

Multimodal AI/ML in cancer research faces challenges such as data availability, computational intensity, overfitting on small datasets (e.g., TCGA), interpretability of black-box models, reproducibility problems due to unpublished code and data, and scalability to enable real-time applications in the clinical setting. Biases arise from institutional differences in staining/scanning, inter-observer annotation discrepancies, selection biases in EHR/omics data, incompletely characterized modalities, and class/demographic imbalances resulting in unfair models. To address these issues, future research should focus on developing standardized benchmarks and interpretable AI for trustworthy precision oncology.

### 11.1 Data quality, harmonization, and heterogeneity

Data incompleteness and noise in datasets such as TCGA and TCIA can be addressed through multimodal fusion, normalization, quality-control procedures, and self-supervised learning approaches. Radiomic and pathomic heterogeneity can be addressed through feature harmonization, cross-modal representation learning, and attention-based integration; however, these approaches may remain sensitive to batch effects and can face scalability challenges when applied to increasingly diverse and large datasets. Sparse datasets alongside staining-related biases are critically highlighted, with potential solutions including distributed data infrastructures and federated learning proposed, but their generalizability remains constrained by the limited size and diversity of available patient cohorts (112). Differences in data formats and acquisition protocols can be addressed through standardized quality control (QC) procedures, data harmonization, and batch-correction methods. However, these preprocessing steps can increase computational and operational complexity and may complicate practical deployment. Challenges associated with the volume, velocity, variety, and veracity of multimodal biomedical data require appropriate normalization, batch correction, and missing data imputation strategies. Methods such as ComBat, DESeq2-based normalization, and graph-based imputation have been explored for addressing specific aspects of these challenges (113). Data silos across diagnostic and clinical settings remain a major barrier to multimodal data integration and can limit the scalability of AI systems across institutions.

### 11.2 Bias, fairness, and representativeness

Through surveys, clinical trials, and ethical analyses, the issues of bias and fairness in multimodal AI for cancer care have been examined. They show that variations may occur in imaging, genomic data, and institutional practices. Recent reviews have emphasized the need for systematic fairness auditing and evaluation across diverse datasets, pointing out that medical imaging and oncology applications lag behind language models (114). Studies indicate that AI performance may vary across demographic and clinical subgroups. In prostate cancer trials, multimodal AI systems gave different results for African and non-African men, showing clear demographic bias in predicting treatment outcomes (115). The multi-omics research indicates that most genomic data come from people of European ancestry, which can make precision cancer medicine less effective for other groups (116). In cancer imaging, AI has been found to diagnose with lower accuracy across demographic groups, prompting experts to recommend fairness checks and transparent reporting (117). To reduce bias, the ethical framework in healthcare AI suggests several practical approaches, such as data reweighting and testing models separately across subgroups, with oncology highlighted as a major concern (118). Early studies in oncology also warned that AI trained primarily in large academic hospitals may not perform well in smaller or rural clinics, leading to unequal access and emphasizing institutional bias (119). Altogether, these studies point to the need for diverse datasets, fairness audits, and robust governance to ensure AI improves cancer care for all patients rather than widening disparities.

In oncology, bias may arise at multiple stages of multimodal data acquisition and model development. Demographic imbalance in training cohorts, including underrepresentation of specific age groups, sexes, ethnicities, or socioeconomic groups, may reduce model generalizability and contribute to inequitable clinical performance (120). Similarly, variations in imaging acquisition protocols, scanner manufacturers, reconstruction algorithms, and staining procedures for whole-slide histopathology images can introduce systematic domain shifts that adversely affect diagnostic accuracy across institutions (121). In multi-omics studies, ancestry imbalance within genomic datasets may bias biomarker discovery and risk prediction models, particularly for underrepresented populations (116). Furthermore, differences in patient populations, clinical workflows, data-collection practices, EHR structures, and case mix across institutions can introduce dataset shift and affect the generalizability of clinical AI models, highlighting the need for multicenter external validation and continuous performance monitoring (120, 122–125).

### 11.3 Model interpretability and clinical trust

AI is increasingly being applied in oncology and pathology; however, limited transparency in how complex models generate predictions remains an important barrier to clinician trust and adoption. Cancer pathology studies indicate that tools such as saliency maps may highlight relevant regions of an image, but they do not necessarily provide explanations that are clinically interpretable or aligned with pathologists’ diagnostic reasoning (126). Disease predictions show that methods such as SHAP and LIME produce mathematical explanations, but they rarely align with oncologists’ reasoning, creating a trust gap (127). Oncology imaging reveals that diagnostic accuracy can vary across groups, and clinicians stress that interpretability must fit naturally into their established workflows (10). Techniques such as Grad-CAM and feature-importance scores may provide visually intuitive explanations but do not necessarily offer clinically actionable information that aligns with oncologists’ reasoning (128). Research in histopathology shows that accuracy alone isn’t enough; clinicians want explanations they can validate against their own expertise (129).

### 11.4 Generalizability and reproducibility

AI models developed in oncology may exhibit reduced performance when evaluated across institutions or patient populations that differ from the development cohort. A major challenge is the limited reproducibility of published AI studies; incomplete reporting of datasets, preprocessing procedures, model architectures, and evaluation protocols can hinder independent reproduction. A recent review on digital pathology and multimodal oncology data points out that the complexity of cancer makes reproducibility especially hard, and selective reporting can distort the interpretation of the available evidence (130). Another paper on AI-driven cancer diagnostics shows that radiology and pathology models often use non-standard pipelines, making it difficult to reproduce results or apply them in new settings (131). Both studies agree that standardized reporting should cover datasets, preprocessing procedures, validation strategies, and even negative results, which is crucial. Multimodal AI cannot be relied on to generalize across a wide range of patients without this transparency, and its clinical significance in oncology will remain limited.

## 12 Ethical, legal, and regulatory considerations

### 12.1 Privacy, security, and data governance

**Figure 4:**
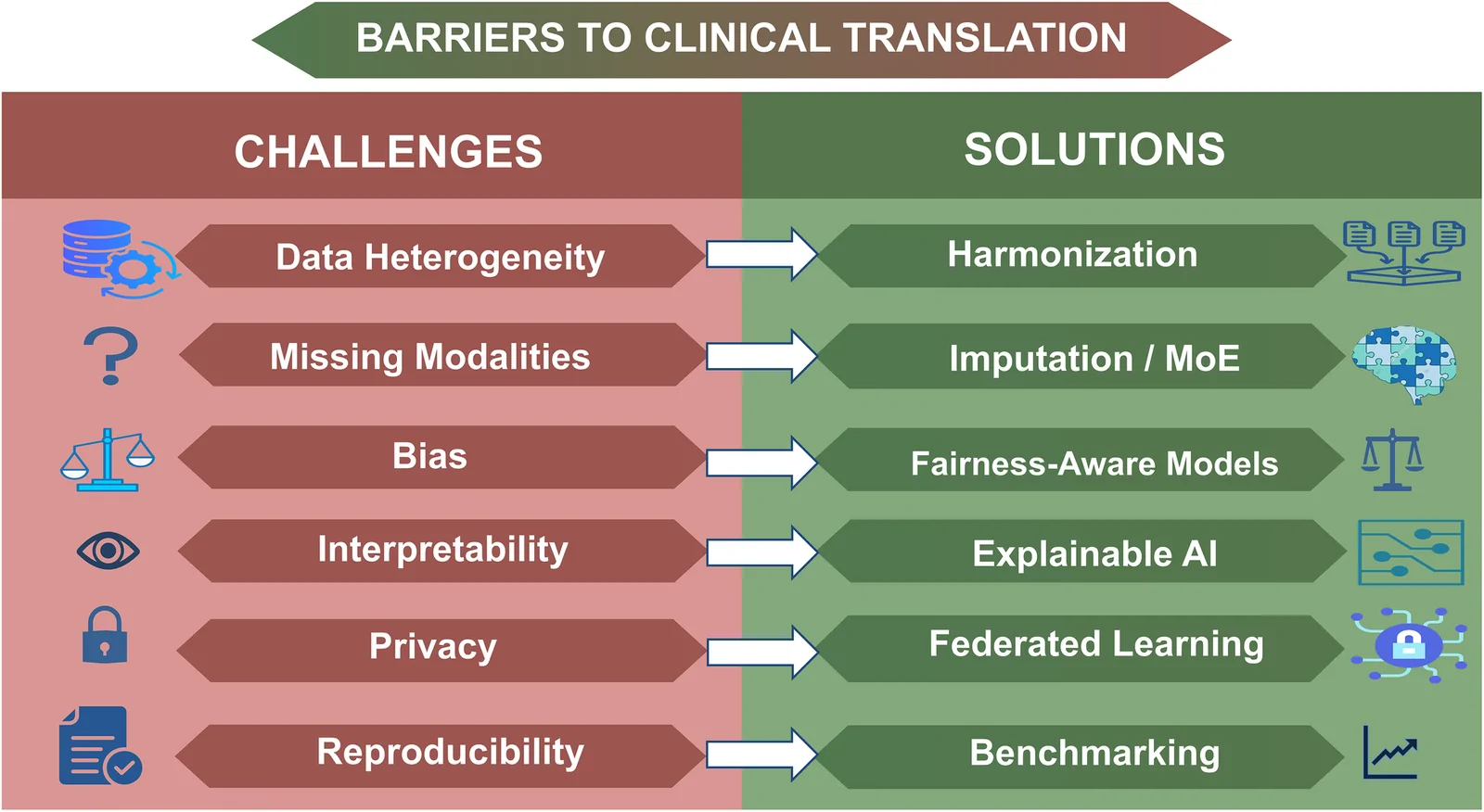
Challenges, biases, and translational solutions in multimodal AI for oncology. This figure summarizes key challenges in multimodal AI for oncology, including data heterogeneity, missing modalities, bias, limited interpretability, reproducibility issues, and privacy concerns. Corresponding solutions such as data harmonization, imputation, fairness-aware modeling, explainable AI, benchmarking, and federated learning are presented. The framework outlines pathways for reliable and scalable clinical translation of multimodal AI systems.

Oncology multimodal data, which integrates imaging, genomics, and clinical records, carries an increased risk of re-identification. Unknown data can also be cross-linked with external sources even without the patient's knowledge, compromising patient confidentiality. Strong governance systems are thus required, which include data minimization, controlled access, and audit trails. Governance should not be limited to technical protection but also to ethical oversight committees and clear consent procedures. The involvement of international partners increases the complexity, as the privacy standards vary across jurisdictions. It is essential to combine the principles of GDPR, HIPAA, and the new AI-related guidelines into a unified approach. Finally, the governance must strike a balance between innovation and patient trust to ensure that multimodal research advances without violating individuals’ rights (132). Key challenges associated with multimodal AI in oncology, along with potential mitigation strategies, are outlined in Figure 4.

### 12.2 Federated and privacy-preserving multimodal learning

FL allows training institutions to collaborate without centralizing sensitive oncology data. FL mitigates the risk of exposure by storing raw data locally, whereas differential privacy (DP) adds statistical noise to further safeguard patient identities. Recent literature indicates that FL + DP models for diagnosing and predicting breast cancer survival and privacy are compatible with predictive accuracy (132). Nevertheless, there are still difficulties: communication overhead, modality limitations, heterogeneity, and poorer model performance when DP is strictly enforced. Bayesian vertical FL methods are being developed to address uncertainty in survival analysis and provide more clinically reliable probabilistic output. These privacy-sensitive measures play a critical role in the scalability of multimodal oncology AI across locations, thereby increasing trust between institutions and regulatory bodies and protecting patients.

### 12.3 Regulatory pathways and evidence standards

Regulatory frameworks for software as a medical device (SaMD) are evolving, particularly for adaptive AI systems that incorporate continuously updated or multimodal inputs. The FDA focuses on lifecycle management, which demands a clear description of the planned use, representative data, and post-market surveillance. The EU's MDR and AI Act also emphasize transparency, bias mitigation, and real-world validation (133). Comparative studies also reflect regional distinctions: the U.S. prefers an iterative approach to change control, with predetermined steps, whereas Europe enforces stricter conformity tests. There is a shift from standards of evidence to dynamic validation, in which performance should be demonstrated across multiple populations and modalities. In oncology, regulators will ask to see evidence that adaptive multimodal systems generalize safely and have mechanisms to detect drift and ensure accountability (134).

### 12.4 Accountability, liability, and medico-legal issues

Attribution of responsibility is complicated when multimodal AI is used to affect clinical decisions. When AI provides survival predictions, liability may depend on whether the system is considered a medical practitioner or a decision-support tool. According to reports, the most feasible models are shared accountability frameworks in which clinicians are ultimately held to account, though developers and institutions are also partially liable. Legal medicine frameworks currently evaluate malpractice lawsuits through the lens of AI, with respect to informed consent, standards of care, and causation. Clinicians also raise concerns about trustworthiness and medico-legal liability, emphasizing the importance of established regulatory demarcations. Finally, accountability should strike a balance between innovation and patient safety, where AI is used to complement human judgment instead of replacing it (135). The major challenges and corresponding mitigation strategies in multimodal AI are summarized in Table 4.

**Table 4:** Challenges, biases, and mitigation strategies in multimodal oncology AI.

Challenge	Source	Impact	Mitigation strategies	References
Data Incompleteness	TCGA and TCIA datasets	Risk of overfitting, interpretability challenges undermining clinical adoption/trust	Multimodal fusion strategies, comprehensive normalization procedures, and self-supervised learning frameworks	(112)
Radiomics and Pathomics Heterogeneity	Batch effects: diverse distribution across modalities	Scalability limitations and difficulty generalizing across large and diverse datasets	Robust feature-alignment and harmonization methods and advanced cross-modal attention mechanisms	(112, 113)
Sparse Datasets and Staining-related biases	Staining variability, small patient cohorts	Restricted generalization and limited robustness of models	High infrastructure and data-storage requirements, federated learning approaches	(112)
Bias and Fairness in Multimodal AI	Variation in imaging, genomic data, institutional practices, demographic imbalance	Unequal performances across demographic groups, reduced effectiveness of precision medicine, and institutional bias	Diverse datasets, fairness audits, transparent reporting, subgroup testing, and ethical frameworks with data reweighing	(114, 115)
Interpretability and Trust Gap	Saliency maps, SHAP, LIME, Grad-CAM, and feature importance scores	Clinicians find explanations misaligned with reasoning, a lack of trust in AI outputs, and workflow incompatibility	Clinically meaningful interpretability methods, fairness checks, transparent reporting, and validation against expert knowledge	(117)
Reproducibility Crisis	Non-standard pipelines, Selective reporting, and lack of shared details	Difficulty reproducing results, misleading evidence, and restricted clinical significance	Standardized reporting protocols, inclusion of negative results, and transparent validation strategies	(131)
Ethical, legal, and regulatory considerations	Risks of re-identification, varying jurisdictional standards, and adaptive AI systems	Compromised patient confidentiality, regulatory complexity, and medico-legal liability concerns	Strong governance systems, GDPR + HIPAA harmonization, consent procedures, lifecycle management, and shared accountability models	(128)
Privacy-preserving federated learning	Communication overhead, modality heterogeneity, DP-induced performance drop	Reduced model accuracy and scalability challenges across institutions	Federated learning with differential privacy, Bayesian vertical FL, and probabilistic outputs for clinical reliability	(132)

## 13 Implementation and health-system integration

### 13.1 Workflow integration and human-AI collaboration

A successful implementation of multimodal AI in oncology processes involves the seamless interaction between human-centered work and machine-generated results. Co-pilot models are increasingly used in tumor boards, where AI synthesizes imaging, pathology, and genomic data to produce concise decision-support summaries. These supplements do not replace oncologists’ deliberations. Interoperability is essential: AI outputs should be integrated with Electronic Health Records (EHRs) and Picture Archiving and Communication Systems (PACS) to avoid disrupting the workflow. Many contemporary PACS environments can be integrated with AI modules for functions such as automated triage, annotation, and audit logging. Plugging AI systems into existing infrastructure relies on standardized data formats (HL7, FHIR) and APIs to enable successful integration. Finally, workflow integration must reduce cognitive load, accelerate tumor board consensus, and make AI information available during natural clinical decision-making (136).

### 13.2 Usability, training, and clinician acceptance

Clinician acceptance depends on usability, transparency, perceived reliability, and compatibility with established clinical workflows. Research indicates that oncologists and radiologists appreciate AI tools that offer clear reasoning, user-friendly interfaces, and compatibility with established platforms. Implementation also requires appropriate training and competency development: AI literacy programs, simulation exercises, and structured onboarding all help clinicians understand the model's limits and how to interpret its outputs responsibly. Change-management strategies, including phased implementation, pilot testing, and structured feedback mechanisms, may facilitate adoption. Surveys show that doctors are more likely to use AI if they believe it will help them do their jobs better rather than replace them. Professional organizations have emphasized the importance of clinician oversight, transparency, and patient-centered implementation of AI in clinical practice. Ultimately, acceptance depends on ensuring that AI aligns with clinical values such as safety, accountability, and improved patient outcomes (137, 138).

### 13.3 Economic evaluation and value-based care

The economic assessment of multimodal AI in oncology emphasizes cost-effectiveness, reimbursement, and equity. Systematic reviews demonstrate that AI reduces costs by eliminating unnecessary procedures, enhancing diagnostic accuracy, and optimizing resource allocation. Incremental cost-effectiveness ratios for oncology AI frequently meet established thresholds, indicating compelling value propositions (137). However, reimbursement models remain inadequate. Payers struggle to determine whether adaptive AI is a diagnostic tool or a clinical service. Value-based care frameworks emphasize outcomes such as quality-adjusted life years (QALYs) and fair access. Policymakers say that AI could make inequalities worse if it is not carefully designed, since it would favor institutions with more resources. There are now clear rules for reporting economic evaluations that decision-makers can use. Ultimately, demonstrating efficiency gains and fair outcomes will be important for securing long-term reimbursement and widespread use (139, 140).

## 14 Emerging trends in multimodal AI for oncology

### 14.1 Foundation models and generative AI

Cancer research involves generating large amounts of data with varying levels of complexity and heterogeneity. Data such as medical images, histopathology slides, molecular profiles, and clinical data are generated in cancer research. Analyzing them individually may not suffice to fully understand the heterogeneous nature of cancer. With recent advances in data analysis, multimodal learning has shown it can improve the accuracy of various applications in cancer research, including but not limited to cancer detection, diagnosis, prediction, and therapy planning (113). Most applications still rely on the individual analysis of each data type. In addition, the conventional machine learning methods are not prepared to handle heterogeneous data types (141). On the other hand, DL has been shown to capitalize on the benefits from the complementarity of different data modalities and to ease the process of representing and fusing heterogeneous data (142). Multimodal learning in oncology refers to the integration of multiple data sources, such as clinical, imaging, and molecular data, that contain different and complementary information. Clinical data reflect the patient's history and the disease course over time (143). The image data reflect the spatial characteristics of the tumors, such as geometry and other features. The molecular data reflect the genes and proteins involved in cancer (129). Multimodal learning can provide a more comprehensive representation of cancer biology and, in selected applications, may improve predictive performance compared with individual modalities; however, the magnitude and consistency of these benefits depend on the data quality, modalities integrated, modeling strategy, and clinical context.

### 14.2 Single-cell, spatial, and longitudinal multimodal data

The ability to profile multiple molecular layers within individual cells at various spatial locations is at the core of recent advancements in single-cell and spatial multi-omics. Modern cellular biology is about studying complex activities occurring across multiple molecular layers, including the genome, epigenome, transcriptome, proteome, and metabolome (144, 145). Single-omics approaches can only provide information about a single characteristic, such as gene expression or gene sequences. A better understanding of the regulation of cellular states, especially during growth, development, aging, and disease, can be achieved by combining multiple molecular layers within and across different molecular modalities (146). Investigating the relationship between genetic variations and their effects on chromatin modifications and gene expression at the single-cell level and across multiple molecular modalities will help uncover the unique molecular mechanisms and cellular dynamics that are missing when analyzing individual modalities (147). Single-cell and tissue engineering, and single-cell and spatial multi-omics, are evolving rapidly, and there is ample scope to improve our understanding of the biology and mechanisms of various diseases (148). While there has been tremendous progress, the research field still has a long way to go. Despite substantial advances, many current methods continue to face trade-offs among spatial resolution, molecular coverage, throughput, and multimodal integration (149). In addition, single-cell multi-omics analysis demands new computational approaches to accurately analyze different omics data (150).

### 14.3 Real-world evidence, learning health systems, and continual learning

The explosion of biomedical data, including clinical datasets, multi-omics data, and imaging, has created numerous opportunities to develop efficient algorithms to facilitate cancer diagnosis, prognosis, and therapy (151). Cancer exhibits substantial heterogeneity across molecular, cellular, and tissue scales, making comprehensive characterization using a single data modality challenging (152). Although individual oncology tasks, such as tumor detection, classification, and prediction using DL models, yield impressive results, unimodal models do not fully leverage the potential of diverse datasets (153). Thus, multimodal DL models that fuse information from heterogeneous datasets have emerged as a valuable strategy for precision oncology (154). Although it is relatively recent, the design and evaluation of such models are supported by the numerous publicly available datasets available for oncology research (155). Such resources provide a rich collection of datasets comprising radiological images, molecular and clinical data, and histopathological images from different cancer types. Examples of such resources include The Cancer Genome Atlas and The Cancer Imaging Archive (156).

### 14.4 Towards clinically deployable multimodal AI ecosystems

There are several specific challenges associated with multimodal oncology data, including limited annotations, privacy concerns, and modality imbalance. DL, which can be used to improve the performance of the analysis of such data, including transfer learning, where pre-trained models are used for learning from the limited data in the clinical environment, and the FL approach, which combines the benefits of large datasets across institutions while keeping the patient data private (132, 157). Different learning paradigms, including supervised, weakly supervised, self-supervised, and unsupervised learning, may be selected according to the availability, quality, and reliability of labeled and unlabeled data (158). Multimodal DL has been well utilized in the cancer care process. For example, multimodal integration of PET, CT, MRI, and histopathology data has been investigated to improve tumor registration and segmentation performance (159). On the other hand, combining imaging data with clinical and genomic information improves cancer detection and diagnosis by enabling more accurate tumor classification and differentiation of tumor subtypes (160).

## 15 Practical roadmap for clinical translation

The successful translation of multimodal AI from research settings to routine oncology practice requires not only advances in computational methodologies but also standardized implementation strategies that ensure robustness, reproducibility, and clinical utility. Fundamental multimodal ML capabilities, including representation, alignment, reasoning, generation, and transfer, provide the computational foundation for integrating heterogeneous biomedical data and generating clinically meaningful insights (161). Representation learning enables informative features to be extracted and integrated across multiple modalities, while multimodal fusion strategies combine complementary information at the input, intermediate, or output level (162). Reasoning facilitates multi-step inference by integrating complementary information across modalities to support diagnosis, prognosis, and treatment decision-making. Furthermore, generative approaches can synthesize new multimodal data, such as generating imaging features from textual descriptions or summarizing complex clinical information, while transference enables knowledge learned from one modality or dataset to improve performance in another, particularly when labeled data are limited (163).

Despite these methodological advances, successful clinical implementation requires a structured translational framework extending beyond algorithm development. Key prerequisites include standardized data acquisition protocols, harmonization of radiological, histopathological, genomic, and clinical datasets, rigorous quality control, prospective multicenter validation, and external testing across diverse patient populations (164). Equally important are interoperability with electronic health record systems and digital pathology platforms, transparent reporting standards, explainable AI frameworks, and compliance with regulatory and ethical requirements. These measures are essential to ensure that multimodal AI systems are reliable, reproducible, and generalizable across different healthcare environments.

Digital pathology represents one of the more advanced areas of multimodal AI research and emerging clinical translation in oncology. Integration of whole-slide histopathology with radiological, genomic, and clinical data has shown potential to improve tumor characterization, molecular subtype classification, prognostic modeling, biomarker discovery, and prediction of therapeutic response in research settings (165). Such multimodal integration provides a comprehensive understanding of tumor biology and supports the development of precision oncology strategies tailored to individual patients.

Robust clinical translation of multimodal AI additionally requires rigorous external validation, model calibration, and prospective clinical evaluation. External validation using geographically and institutionally independent datasets is essential to assess model generalizability beyond the original development cohort. Calibration analysis ensures that predicted probabilities accurately reflect observed clinical outcomes, thereby improving the reliability of risk estimation and treatment decision-making. Furthermore, dataset shift arising from temporal changes, evolving clinical practice, differences in patient demographics, or institutional variations may substantially reduce model performance after deployment. Consequently, prospective multicenter studies, continuous model monitoring, and periodic recalibration should be considered essential prerequisites for the routine integration of multimodal AI systems into clinical oncology workflows (166, 167).

Nevertheless, several challenges continue to impede widespread clinical adoption. The availability of large, well-annotated multimodal datasets remains limited, restricting model generalizability across institutions and patient populations. Many AI algorithms also function as “black-box” models, limiting clinical interpretability and reducing physician confidence in automated decision-making (168). In addition, inadequate digital infrastructure, lack of standardized workflows, limited technical expertise, and disparities in healthcare resources present substantial barriers, particularly in low- and middle-income settings. Ethical, legal, and regulatory considerations, including data privacy, security, algorithmic bias, fairness, and accountability, must also be systematically addressed before multimodal AI systems can be safely integrated into routine oncology practice (169). Continued collaboration among clinicians, computational scientists, regulatory agencies, and healthcare organizations will therefore be essential to establish standardized benchmarks, promote prospective clinical validation, and facilitate the responsible implementation of multimodal AI for precision cancer care.

## 16 Conclusion

Multimodal AI represents a transformative advancement in precision oncology by enabling the integration of complementary data modalities, including medical imaging, digital pathology, multi-omics, electronic health records, and patient-reported outcomes. Compared with unimodal approaches, multimodal AI can provide a more comprehensive representation of tumor biology and has shown potential across cancer screening, diagnosis, molecular profiling, prognostic assessment, treatment selection, radiotherapy planning, and longitudinal patient management. Recent developments in GNNs, transformer-based architectures, and foundation models have further enhanced multimodal AI systems’ ability to learn complex cross-modal representations and to support personalized cancer care. Despite these advances, the widespread clinical adoption of multimodal AI remains constrained by several scientific, technical, and regulatory challenges. The availability of large, diverse, and well-annotated multimodal datasets, together with standardized data acquisition and harmonization protocols, remains fundamental for developing robust and generalizable models. In addition, external and prospective multicenter validation, transparent reporting, reproducible model development, fairness auditing, explainability, and continuous performance monitoring are essential to ensure reliable deployment across diverse clinical settings. Integration with existing healthcare infrastructure, including electronic health records and picture archiving and communication systems, as well as compliance with evolving ethical, legal, and regulatory frameworks, will be equally important for responsible clinical implementation. Overall, multimodal AI has the potential to fundamentally reshape precision oncology by supporting more accurate, personalized, and data-driven cancer care. Realizing this potential will require sustained multidisciplinary collaboration among clinicians, computational scientists, healthcare institutions, industry partners, and regulatory agencies to establish standardized benchmarks, validate models across diverse populations, and develop trustworthy AI systems that are safe, equitable, interpretable, and readily deployable in routine oncology practice.
